# Abstract Computation in Schizophrenia Detection through Artificial Neural Network Based Systems

**DOI:** 10.1155/2015/467178

**Published:** 2015-03-05

**Authors:** L. Cardoso, F. Marins, R. Magalhães, N. Marins, T. Oliveira, H. Vicente, A. Abelha, J. Machado, J. Neves

**Affiliations:** ^1^University of Minho, CCTC, 4710-057 Braga, Portugal; ^2^Life and Health Sciences Research Institute (ICVS), School of Health Sciences, University of Minho, 4710-057 Braga, Portugal; ^3^ICVS/3B's, PT Government Associate Laboratory, Guimarães, 4710-057 Braga, Portugal; ^4^Centro Hospitalar Conde de Ferreira, 4200-227 Porto, Portugal; ^5^Department of Chemistry and Évora Chemistry Centre, School of Science and Technology, University of Évora, 7004-516 Évora, Portugal

## Abstract

Schizophrenia stands for a long-lasting state of mental uncertainty that may bring to an end the relation among behavior, thought, and emotion; that is, it may lead to unreliable perception, not suitable actions and feelings, and a sense of mental fragmentation. Indeed, its diagnosis is done over a large period of time; continuos signs of the disturbance persist for at least 6 (six) months. Once detected, the psychiatrist diagnosis is made through the clinical interview and a series of psychic tests, addressed mainly to avoid the diagnosis of other mental states or diseases. Undeniably, the main problem with identifying schizophrenia is the difficulty to distinguish its symptoms from those associated to different untidiness or roles. Therefore, this work will focus on the development of a diagnostic support system, in terms of its knowledge representation and reasoning procedures, based on a blended of Logic Programming and Artificial Neural Networks approaches to computing, taking advantage of a novel approach to knowledge representation and reasoning, which aims to solve the problems associated in the handling (i.e., to stand for and reason) of defective information.

## 1. Introduction

Schizophrenia is a brain disorder that strikes people as they are entering the prime of their life and, in many cases, may run a recurrent and ultimately chronic course that will lead to substantial disability. Indeed, this highly destructive mental illness affects the essence of what makes people human, that is, their personality and intellect [1].

The incidence of this disease is roughly one new patient per year per ten thousand human beings. In the one hand it occurs in all cultures, in spite of more schizophrenic ones appearing in the lower socioeconomic classes of the industrialized countries; on the other hand, the admission rates for schizophrenia are higher in the urban areas than in the rural ones. The gender distribution is approximately equal in both sexes, and the peak of incidence is situated between 15 and 25 years in males, and between 25 and 35 years in females [1, 2].

There are also several risk factors that must be considered, namely, the genetic ones like predisposing, pregnancy, and birth complications. Indeed, and to some extent, some precipitating circumstances, such as family interactions (e.g., dysfunctional families and expressed emotion, a kind of behavior with overt criticism and hostility), life events (e.g., happenings that provokes a high level of stress), or drug abuse, must also be object of attention [2].

When working on the diagnosis of schizophrenia, the challenging question with which practitioners are met relies on the similarity between the signs and symptoms among psychic diseases and states. Typically, the earliest act for the diagnosis of schizophrenia proceeds from the detection of a specific outbreak, ordinarily evinced by hallucinations, delusions, disorganized speech, catatonic behavior, abolition, and social separation or even additional personality disorders. The psychiatric diagnosis is made through the clinical interview based on psychopathology, but the problem is that knowledge of psychopathology of anthology depends on the training and experience of the psychiatrist. These limitations “open the door” to mistake and doubt. From here, the practitioners may perform psychic exams intended to detect cognitive flaws, delusional perception, and abnormal changes or thoughts, among others [1, 2].

Above any kind of dispute, the main problem with the diagnosis of schizophrenia comes from the large number of different mental diseases and states that may mimic its signs, such as epilepsy, drug-induced psychosis, affective psychoses (e.g., bipolar, major depressive disorders), Asperger syndrome, schizoaffective disorder, and Wilson's disease. To face this situation the practitioner is forced to order extra exams, namely, toxicological tests, electroencephalography (EEG) and brain computed tomography (CT)'s [1]. Also this pinpointing is usually done over a large period of time (at least 6 months), therefore generating a huge amount of data, which has to be treated and interpreted by the practitioner(s).

Facing such a large amount of facts, even experienced psychiatrists have difficulties to make a precise diagnosis and distinguishing between this and other diseases. Unfortunately, there is not too much work done in the area of Medical Informatics to help in this process, although Strous et al. [3] worked on a system that uses the differences on writing to diagnose schizophrenia.

With this paper we make a start on the development of an unusual or original diagnosis assistance system for schizophrenia. We will present a logic programming based approach in order to represent the knowledge and reasoning, with a focus on the Degree of Confidence (DoC) of the attributes set, that makes a function or a predicate [4].

## 2. Related Work

Many studies presenting the concept of uncertainty and/or “imperfect data” like [5–8] show that there is an emergent interest in the problem of uncertainty as compared to accuracy or error in data [9, 10]. The notion of uncertainty is broader than error or accuracy and includes these more restricted concepts. While accuracy is the closeness of measurements or computations to their “true” value or some value agreed to be the “truth”, uncertainty can be considered any aspect of the data that results in less than perfect knowledge about the phenomena being studied [11–13].

The consequences of the uncertainty in the data and in the data quality to user exploring, modelling, visualizing, and querying have been also referred to in many studies [6, 8, 14–18]. On the one hand, it is consensual that when the data are uncertain, a different representation and uncertainty which can be reduced by “acquiring additional information or improving the quality of the information available” [6] is needed; that is, in almost all decisions that one may take, the information is not always exact, but indeed imperfect, in the sense that we handle estimated values, probabilistic measures, or degrees of uncertainty [19, 20]. On the other hand, knowledge and belief are generally incomplete, contradictory, or even error sensitive. It is advisable the use of formal tools to deal with the problems that arise with the use of partial, contradictory, ambiguous, imperfect, nebulous, or missing information [13, 21–23]. Some general models have been presented [8, 16, 24], where uncertainty is associated to the application of Probability Theory [16, 17], Fuzzy Set Theory [25], Similarities [26, 27]. Other approaches for knowledge representation and reasoning have been proposed using the Logic Programming (LP) paradigm, namely, in the area of Model Theory [28–30] and Proof Theory [29, 31, 32]. Qualitative models and qualitative reasoning have been around in Artificial Intelligence research for some time [14, 33], in particular due to the growing need to offer support in decision-making processes. The evaluation of knowledge that stems out from logic programs becomes a point of research. In this sense, the evaluation of knowledge that stems out from logic programs becomes a point of research. Shi et al. [15] and Schneider [25] work is a good example of quality evaluation using logic. They used abduction [14] and temporal logic for quality-checking of medical guidelines, proposing a method to diagnose potential problems in a guideline, regarding the fulfillment of general medical quality criteria at a metalevel characterization. They explored an approach which uses a relational translation to map the temporal logic formulas to first-order logic and a resolution-based theorem prover [25]. In another research line, the Quality-of-Information concept (QoI) [6, 7, 26, 34] demonstrated their applicability in many dynamic environments and for decision making purposes. The objective is to build a quantification process of the QoI and an assessment of the argument values of a given predicate with relation to their domains (here understood as Degree-of-Confidence (DoC)), which stems from a logic program or theory during the evolutive process of searching solutions in order to solve a problem in environments with default data. Our main contribution relies on the fact that at the end, the initial extensions of the predicates that make the universe of discourse are given in terms of DoCs predicates that stand for one's confidence that the initial predicates arguments values of the predicates that make the universe of discourse fit into their respective domains. This approach potentiates the use of diverse computational paradigms, like Case Based Reasoning [35], Artificial Neural Networks (ANN) [36, 37], and Particle Swarm [38], just to name a few. It also encapsulates, in itself a new vision of Multivalue Logics, once a proof of a theorem in a conventional way is evaluated to the interval [0,1]. Following these studies good results were achieved for different purposes, namely, in Healthcare [27], Civil Law [39], Multiagent Systems [40], Virtual Entities [34], Ambient Assisted Living [41], and Decision Making Environments [30]. Indeed, some interesting results have been obtained, namely, in the fields of Coronary Risk Evaluation [42], Hyperactivity Disorder [43], and Length of Hospital Stay [44] among others, which fit the case studies insufficiency that are common in this area of research and applications.

## 3. Knowledge Representation and Reasoning

We follow the proof theoretical approach and an extension to the LP language [31], to knowledge representation and reasoning. An Extended Logic Program (ELP) stands for a finite set of clauses in the form:

(1) $$\begin{array}{c} q⟵p_{1}\wedge p_{n}\wedge \text{not}q_{1}\wedge \cdots \wedge \text{not}q_{m}\\ ?\left(p_{1}\wedge \cdots \wedge p_{n}\wedge \text{not}q_{1}\wedge \cdots \wedge \text{not}q_{m}\right)\;\left(n,m\geq 0\right), \end{array}$$

where ? is a domain atom denoting falsity and the *p*_*i*_, *q*_*j*_, and *p* are classical ground literals, that is, either positive atoms or atoms preceded by the classical negation sign ¬ [32]. Under this representation formalism, every program is associated with a set of abducibles [28, 30], given here in the form of exceptions to the extensions of the predicates that make the program. Once again, LP has emerged as an attractive formalism for knowledge representation and reasoning tasks, introducing an efficient search mechanism for problem solving. Due to the growing need to offer user support in decision-making processes some studies have been presented [45, 46], related to the qualitative models and qualitative reasoning in Database Theory and in Artificial Intelligence (AI) research. With respect to the problem of knowledge representation and reasoning mechanisms in LP, a measure of the Quality of Information (QoI) of such programs has been object of some work with promising results [47–49], that is, the QoI denotes one's confidence that a particular term of a predicate that makes the universe of discourse belongs to its extension. Indeed the QoI indeed [31] with respect to the extension of a predicate *i* will be given by a truth-value in the interval [0,1]; that is, if the information is known (positive) or false (negative) the QoI for the extension of predicate *i* is 1. For situations where the information is unknown, the QoI is given by

(2) $$\begin{array}{c} {\text{QoI}}_{i}=\underset{N\to \infty }{\lim}\frac{1}{N}=0\;\left(N\gg 0\right), \end{array}$$

where *N* denotes the cardinality of the set of terms or clauses of the extension of predicate *i* that stand for the incompleteness under consideration. For situations where the extension of predicate *i* is unknown but can be taken from a set of values, the QoI is given by

(3) $$\begin{array}{c} {\text{QoI}}_{i}=\frac{1}{\text{Card}}, \end{array}$$

where Card denotes the cardinality of the abducibles set for *i*, if the abducibles set is disjoint. If the abducibles set is not disjoint, the QoI is given by

(4) $$\begin{array}{c} {\text{QoI}}_{i}=\frac{1}{\left(C_{1}^{\text{Card}}+\cdots +C_{\text{Card}}^{\text{Card}}\right)}, \end{array}$$

where *C*_Card_^Card^ is a card-combination subset, with Card elements. The next element of the model to be considered is the relative importance that a predicate assigns to each of its attributes under observation, that is, *w*_*i*_^*k*^, which stands for the relevance of attribute *k* in the extension of predicate_*i*_. It is also assumed that the weights of all the attribute predicates are normalized, that is

(5) $$\begin{array}{c} \underset{1\leq k\leq n}{\sum }w_{i}^{k}=1,\;\forall_{i}, \end{array}$$

where ∀ denotes the universal quantifier. It is now possible to define a predicate_*i*_ scoring function *V*_*i*_(*x*) so that, for a value *x* = (*x*_1_,…, *x*_*n*_), defined in terms of the attributes of predicate_*i*_, one may have

(6) $$\begin{array}{c} V_{i}\left(x\right)=\underset{1\leq k\leq n}{\sum }w_{i}^{k}\times \frac{{\text{QoI}}_{i}\left(x\right)}{n}. \end{array}$$

It is now feasible to rewrite the extensions of the predicate referred to above, according to productions of the type

(7) $$\begin{array}{c} {\text{predicate}}_{i}\left(x_{1},\ldots ,x_{n}\right)\;\text{::}\;\text{QoI}\;\text{::}\;\text{DoC} \end{array}$$

and evaluate the Degree of Confidence (DoC) given by DoC = *V*_*i*_(*x*_1_,…, *x*_*n*_)/*n* which denotes one's confidence that the argument values of predicate_*i*_ fit into its domain values. To be more general, let us suppose that the Universe of Discourse is described by the extension of the predicates:

(8) $$\begin{array}{c} a_{1}\left(\cdots \right),a_{2}\left(\cdots \right),\ldots ,a_{n}\left(\cdots \right)\;\left(n\geq 0\right). \end{array}$$

Therefore one may have (where ⊥ denotes an argument value of the type unknown; the values of the others arguments stand for themselves):

(9) ¬a1x1,y1,z1⟵not  a1x1,y1,z1a1⊥,10,20,15 :: 1 :: DoC5,10 5,30 10,20︸attributes's  domains  for  x1,y1,z1attributes's  dom⋮¬a2x2,y2,z2⟵not  a2x2,y2,z2a245,54,10,12,⊥ :: 1 :: DoC30,60 6,14 2000,6000︸attributes's  domains  for  x2,y2,z2attributes's  dom⋮⇓1st  interaction:  transition  to  continuous  intervals¬a1x1,y1,z1⟵not  a1x1,y1,z1a15,10,10,20,15 :: 1 :: DoC5,10 5,30 10,20︸attributes's  domains  for  x1,y1,z1attributes's  dom⋮¬a2x2,y2,z2⟵not  a2x2,y2,z2a245,54,10,12,2000,6000 :: 1 :: DoC30,60 6,14 2000,6000︸attributes's  domains  for  x2,y2,z2attributes's  dom⋮⇓2nd  interaction:  normalization:  Y−Ymin⁡Ymax⁡−Ymin⁡¬a1x1,y1,z1⟵not  a1x1,y1,z1a15−510−5,10−510−5,10−530−5,20−530−5,llllll15−1020−10,15−1020−10 ≡a10,1,0.2,0.6,0.5,0.5 :: 1 :: DoC0,1 0,1 0,1︸attributes's  domains  for  x1,y1,z1attributes's  dom⋮¬a2x2,y2,z2⟵not  a2x2,y2,z2a245−3060−30,54−3060−30,10−614−6,12−614−6,lllllll2000−20006000−2000,6000−20006000−2000 ≡a20.5,0.8,0.5,0.75,0,1 :: 1 :: DoC0,1 0,1 0,1︸attributes's  domains  for  x2,y2,z2attributes's  dom⋮.

**Figure 1:**
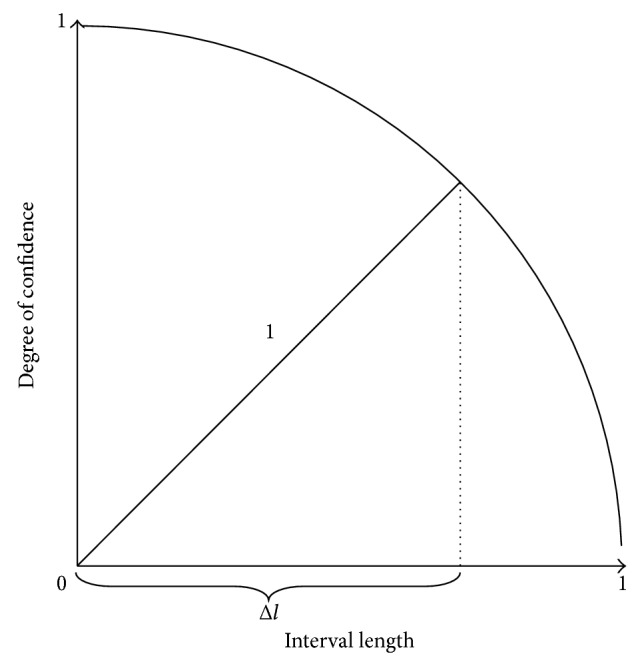
Evaluation of the Degree of Confidence.

The Degree of Confidence (DoC) is now evaluated using $\text{DoC}=\sqrt{1-\Delta l^{2}}$, as it is illustrated in Figure 1. Here Δ*l* stands for the length of the arguments intervals, once normalized.

As shown in Figure 1, one has the expected representation of the universe of discourse, where all the predicates' arguments are nominal. They speak for one's confidence that the unknown values of the arguments fit into the correspondent intervals referred to above

(10) ¬a1DoCx1,y1,z1⟵not  a1DoCx1,y1,z1a1DoC0,0.916,1 :: 0.5 :: DoC0,1 0.2,0.6 0.5,0.5︸attributes's  values  range  for  x1,y1,z10,1 0,1 0,1︸attributes's  domain  for  x1,y1,z1attributes's  dom⋮¬a2DoCx2,y2,z2⟵not  a2DoCx2,y2,z2a2DoC0.954,0.968,0 :: 0.6 :: 0.6410.5,0.8 0.5,0.75 0,1︸attributes's  values  ranges  for  x2,y2,z20,1 0,1 0,1︸attributes's  domain  for  x2,y2,z2attributes's  dom⋮.

## 4. One's Model

Therefore, and in order to exemplify the applicability of our approach to default knowledge, we will look at the relational database model, since it provides a basic framework that fits into our expectations [50] and is understood as the genesis of the LP approach to knowledge representation and reasoning.

**Figure 2:**
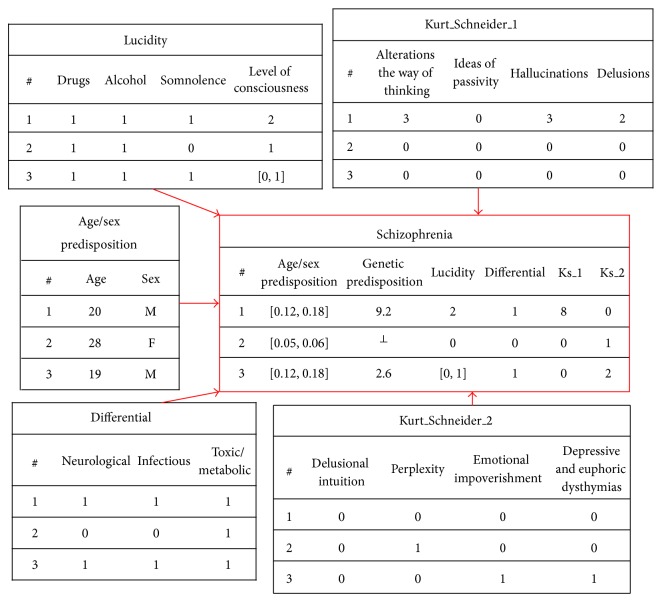
An extension of the relational database model.

Consider the scenario where a relational database is given in terms of the extensions of the relations or predicates depicted in Figure 2, which stands for a situation where one has to manage information about schizophrenia disease detection. Under this scenario some incomplete data is also available. For instance, to patient “2” the genetic predisposition value is unknown, while to patient “3” the lucidity (in both the extensions of the schizophrenia relation or predicate) is in the interval [0,1], although the domain values to genetic predisposition range in the interval [0.9,91.5], and the ones to lucidity are bound by the interval [0,2].

The psychiatrist fills the tables that link to the schizophrenia table while he/she executes the psychopathologic exam. Some symptoms may be detected by the psychiatrist; others may be perceived by additional exams (e.g., this happens with some of the attributes of the Differential/Lucidity table). At this point the psychiatrist may fill some attributes with the unknown symbol (⊥) and update the tables later.

The age/sex predisposition parameter, which is evidenced in the schizophrenia table in Figure 2, is based on Table 1 adapted from [1]. These predisposition values are clustered by age group and sex. Thus, the domain for this parameter is in the range [0,0.18] for males, and for females is in the range [0,0.07]. The genetic predisposition parameter, which is also evidenced in the schizophrenia table in Figure 2, is based on Table 2 adapted from [51]; its domain is in the range [0.9,91.5].

Lucidity is given in terms of the parameters or arguments that make a piece of Lucidity Table (Figure 2). The first three parameters are populated with a value between [0,1], wherein 0 (zero) denotes the presence of drugs and 1 (one) the absence of drugs, alcohol or somnolence. The level of consciousness ranges in the interval [0,2], wherein zero means that the patient is in a torpor state; one means that the patient presents obnubilation of the consciousness; and two means he is in a waking state. The differential diagnoses are represented in the differential table of the (Figure 2). Like the table lucidity, it is populated with values between [0,1], meaning the presence or absence of neurological, infectious or toxic/metabolic diseases. The lucidity and the differential diagnoses are important factors in the schizophrenia diagnosis, because if the patient is not lucid or he/she has one of the diseases previously mentioned, the diagnosis is compromised. In other words, if one of the parameters of the lucidity and differential tables is zero, the diagnosis is not settled properly. Thus, the lucidity and differential values in the schizophrenia table are calculated through the multiplication of each parameter of table lucidity by the ones of the differential one. In this way the domain of lucidity parameter in the schizophrenia table is in the range [0,2], while the domain to the differential value is in the range [0,1].

The tables Kurt_Schneider_1 and Kurt_Schneider_2 represent the schizophrenia symptoms of first and second order delineated by Kurt Schneider [1, 2]. Relative to the table Kurt_Schneider_1 (Ks_1) there are four clusters of symptoms. The former one, alterations to the way of thinking, ranges in the interval [0,3], with the meaning: 0 (zero), absence of alterations; 1 (one), obsessive ideas; 2 (two), escape of ideas; 3 (three), loudness, theft, broadcasting, insertion, interruption, and diffusion of the thinking. The ideas of passivity are categorized in the interval [0,2], with the connotation: (0) zero, absence of feelings, impulses or somatic sensations arising from outside that imposed on the patient; 1 (one), feelings, impulses or psychomotor activities with constant doubt or uncertainty; 2 (two), feelings, impulses, psychomotor activities, or somatic sensations strange to himself and controlled by external agents to himself. Hallucinations are rated in the interval [0,4], with the connotation: 0 (zero), absence of hallucinatory phenomena; 1 (one), elementary hallucinations; 2 (two), visual hallucinations; 3 (three), gustatory, olfactory, and kinesthetic hallucinations; 4 (four), auditory hallucinations. The latest, delusions, is grouped in the interval [0,2]: 0 (zero), absence of delusional ideas; 1 (one), delusional ideas assigning meanings of affective basis; 2 (two), delusional perception assigning a new meaning of autoreferential way. In Kurt_Schneider_2 (Ks_2) table all symptoms, showed in the (Figure 2), range in the interval [0,1], wherein 0 (zero) denotes the absence of the symptom and 1 (one) its presence. Once these two tables are populated, the values of Ks_1 and Ks_2 in the schizophrenia table are the sum of the values of respective symptoms. In this way these values range in the intervals [0,11] for Ks_1, and [0,4] for Ks_2. The higher these values are, more evident is the disease. It is of great significance to note that first order symptoms have a much higher influence than the second order ones in schizophrenia detection.

Let us now consider the extensions of the relations given in (Figure 2) to populate the extension of the schizophrenia predicate:

(11) schizophrenia:  Age/Sex Predisposition,Genetic Predisposition,schizophihre.:  Lucidity,Differential,Ks_1,Ks_2⟶0,1,

where 0 (zero) and 1 (one) denote, respectively, the truth-values false and true. It is now possible to give the extensions of the predicate referred to above, which may be set in the form:

(12) ¬schizophreniaAS(predisposition),G(predisposition),Luc(idity),llllllllllllllllllllllDif(ferential),Ks_1,Ks_2AS(predisposition)⟵not  schAS,G,Luc,Dif,Ks_1,Ks_2sch0.12,0.18,9.2,2,1,8,0 :: 1 :: DoC0,0.18 0.9,91.5 0,2 0,1 0,11 0,4︸attribute's  values  rangessch0.05,0.06,⊥,0,0,0,1 :: 1 :: DoC0,0.07 0.9,91.5 0,2 0,1 0,11 0,4︸attribute's  values  rangessch0.12,0.18,2.6,0,1,1,0,2 :: 1 :: DoC0,0.18 0.9,91.5 0,2 0,1 0,11 0,4︸attribute's  values  ranges.

In this program, the first clause denotes the closure of predicate schizophrenia. The next three clauses correspond to the three cases presented in the extension of the schizophrenia relation in (Figure 2). For example, the second clause corresponds to the patient 2 (two), a 28 (twenty eight) years old woman. Analyzing (Table 1), the psychiatrist fills its age/sex predisposition within the interval [0.05,0.06], face a domain that ranges between [0,0.07]. Looking at its genetic predisposition, the psychiatrist does not have any information. In this case the symbol “⊥” is used, which stands for an unknown value. It subsumes that this variable may take any value according to its domain, that is, in the range ([0.9,91.5]).

It is now possible to have the arguments of the predicates extensions normalized to the interval [0,1], in order to compute one's confidence that the nominal value of the arguments under considerations fit into the intervals depicted previously. The normalization process presented below is with respect to patient 2 (two).

Consider

(13) ¬schizophreniaAS(predisposition),G(predisposition),Luc(idity),llllllllllllllllllllllDif(ferential),Ks_1,Ks_2AS(predisposition)⇓sch0.05,0.06,⊥,0,0,0,1 :: 1 :: DoC0,0.07 0.9,91.5 0,2 0,1 0,11 0,4︸attribute's  domain⇓1st  interaction: transition  to  continuous  intervalssch0.05,0.06,0.9,91.5,0,0,0,0, lllllll0,0,1,1:: 1 :: DoC0,0.07 0.9,91.5 0,2 0,1 0,11 0,4︸attribute's  domain⇓2nd  interaction:  normalization:  Y−Ymin⁡Ymax⁡−Ymin⁡sch0.71,0.86,0,1,0,0,0,0,0,0, llllll0.25,0.25:: 1 :: DoC0,1 0,1 0,1 0,1 0,1 0,1︸attribute's  domains⇓DoC  calculation: : DoC=1−Δl2schDoC0.989,0,1,1,1,1 :: 1 :: 0.830.71,0.86 0,1 0,0 0,0 0,0 0.25,0.25︸attribute's  values  range0,1 0,1 0,1 0,1 0,1 0,1︸attribute's  domains.

**Figure 3:**
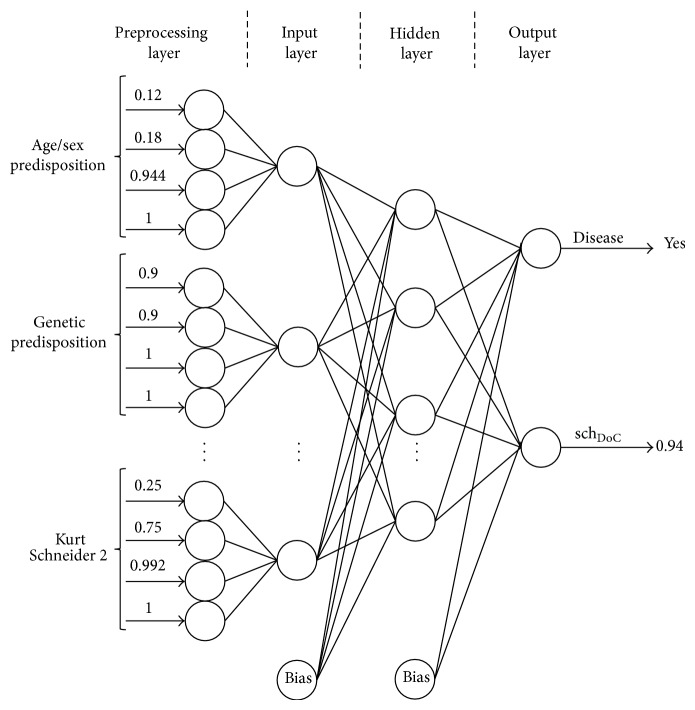
The ANN for the 22-year-old male.

Its terms make the training and test sets of the Artificial Neural Network given below (Figure 3).

## 5. The Artificial Neural Network Topology

The previously presented diagnosis model works well to demonstrate how all the information comes together to form a diagnosis, but it was built with the pure objective of demonstration. In this section, more reliable ways to assemble this information are considered. Different Artificial Intelligence (AI) and data mining tools can be used. Neves et al. [36, 37] demonstrated how a Artificial Neural Network (ANN) can be successfully used to model data and capture complex relationships between inputs and outputs. ANNs simulate the structure of the human brain being populated by multiple layers of neurons. The knowledge representation model defined in this work is appropriate for use with ANNs.

Using a real case of 22-year-old male patient with no schizophrenic relatives, an experienced psychiatrist, during the diagnosis elaboration, verified a full lucidity state of the patient (drugs: 1; alcohol: 1, somnolence: 1; level of consciousness: 2). In the differential diagnoses the symptoms verified were: neurological: 1; infectious: 1; toxic/metabolic: ⊥ (it was not possible to detect). The patient also presented: theft, broadcasting, and diffusion of the thinking; absence of feelings, impulses or somatic sensations arising from outside that imposed on the patient; absence of hallucinatory phenomena; delusional perception assigning a new meaning of autoreferential way. Therefore, the values inserted in the Ks_1 table were: alterations the way of thinking: 3; ideas of passivity: 0; Hallucinations: 0; Delusions: 2. Regarding the Kurt Scheneider symptoms of second order, the psychiatrist detected a weak presence of emotional impoverishment symptoms and the absence of the other three symptoms (delusional intuition: 0; perplexity: 0; emotional impoverishment: [0.25,0.75]; depressive and euphoric dysthymias: 0). In this way, the schizophrenia clauses obtained are sch([0.12,0.18], 0.9,2, ⊥, 5, [0.25,0.75]) :: 1 :: DoC and sch_DoC_(0.944,1, 1,0, 1,0.992) :: 1 :: 0.82. In (Figure 3) it is shown how the normalized values of the interval extremes and theirs DoC values work as inputs to the ANN. The output translates the chance of having schizophrenia and the DoC that one has on such a result. In addition, it was built a database of study cases that were used to train and test the ANNs. As (Figure 3) demonstrates, it was detected schizophrenia in the patient with a DoC = 0.94.

## 6. Conclusions

The diagnosis of schizophrenia is a hard and complex task which needs to consider many different conditions with intricate relations among them. These characteristics put this problem into the area of problems that may be tackled by AI techniques. Despite that, little to no work has been done in that direction.

In this work the founding of a computational framework that uses powerful knowledge representation techniques to represent and structure the information. is presented. This representation is above everything else, very versatile and capable of covering every possible instance representation by considering incomplete and unknown data. This finding has several reasons, namely, that data is not equal to information; the translation of the raw measurements into interpretable and actionable read-outs is challenging; and read-outs can deliver markers and targets candidates without preconception; that is, knowing how human conditions and risk factors may affect schizophrenia detection.

## 7. Future Work

The knowledge representation and reasoning techniques presented above are very versatile and capable of covering every possible instance by considering incomplete, contradictory, and even unknown data. Indeed, the new paradigm for knowledge representation and reasoning enables one to use the normalized values of the interval boundaries and their DoC values, as inputs to different computational paradigms. The output translates the problem solution and the systems confidence on such a happening. Indeed the same problem must be approached using other computational frameworks like Case Based Reasoning [35] or Particle Swarm [38], among others.

## Figures and Tables

**Table 1:** Age/sex predisposition, adapted from [1].

Age/sex predisposition		
(Annual rate per 1000)		
Age group	Male	Female
0–14	[0, 0.18]	[0, 0.07]
15–24	[0.12, 0.18]	[0.06, 0.07]
25–34	[0.06, 0.12]	[0.05, 0.06]
35–44	[0.03, 0.06]	[0.03, 0.05]
45–54	[0.02, 0.03]	[0.03, 0.04]
55–64	[0.01, 0.02]	[0.02, 0.04]
>65	[0, 0.01]	[0, 0.02]

**Table 2:** Genetic predisposition, adapted from [51].

Genetic predisposition (%)	
General population	0.9
Foster brothers	1.8
Spouses	2.1
First cousins	2.6
Nieces and nephews	3.9
Grandchildren	4.3
Stepbrothers	7.1
Parents	9.2
Brothers	14.2
Dizygotic twin	14.5
Dizygotic twin of the same sex	17.6
Sons and daughters	16.4
Children of father and mother schizophrenics	39.2
Monozygotic twin living separately	77.6
Monozygotic twin living together	91.5
